# Similarity-based link prediction in social networks using latent relationships between the users

**DOI:** 10.1038/s41598-020-76799-4

**Published:** 2020-11-18

**Authors:** Ahmad Zareie, Rizos Sakellariou

**Affiliations:** Department of Computer Science, The University of Manchester, Manchester, M13 9PL UK

**Keywords:** Engineering, Mathematics and computing

## Abstract

Social network analysis has recently attracted lots of attention among researchers due to its wide applicability in capturing social interactions. Link prediction, related to the likelihood of having a link between two nodes of the network that are not connected, is a key problem in social network analysis. Many methods have been proposed to solve the problem. Among these methods, similarity-based methods exhibit good efficiency by considering the network structure and using as a fundamental criterion the number of common neighbours between two nodes to establish structural similarity. High structural similarity may suggest that a link between two nodes is likely to appear. However, as shown in the paper, the number of common neighbours may not be always sufficient to provide comprehensive information about structural similarity between a pair of nodes. To address this, a neighbourhood vector is first specified for each node. Then, a novel measure is proposed to determine the similarity of each pair of nodes based on the number of common neighbours and correlation between the neighbourhood vectors of the nodes Experimental results, on a range of different real-world networks, suggest that the proposed method results in higher accuracy than other state-of-the-art similarity-based methods for link prediction.

## Introduction

Social networks are getting lots of attention to capture people’s interactions, partly as a result of the increased use of social media platforms. The large amount of data that may be associated with social networks has motivated research in a number of topics. Among these topics, the identification of missing links and prediction of future links is an important branch of social network analysis^1^. Link prediction is defined as the estimation of the likelihood of link formation between each pair of nodes for which a link does not exist. It has applications in a number of areas, such as, prediction of evolution in dynamic networks^2^, providing recommendation for friends in social networks^3^, finding latent links in an area of concern for security^4^, or finding missing links in networks^5,6^.

Different methods for the link prediction problem have been proposed^4,7^. In similarity-based methods^8–11^, structural similarity between a pair of nodes is taken into account to estimate the probability of link formation between the nodes. Nodes with high similarity tend to have a future relationship. Conversely, in probabilistic methods^12,13^, information beyond structure, such as behaviour of users and link features are required. However, the lack of sufficient and/or accurate information^4^ about such features has motivated researchers to focus primarily on similarity-based methods and how to estimate structural similarity from which the likelihood of link formation between each pair of nodes can be derived.

**Figure 1:**
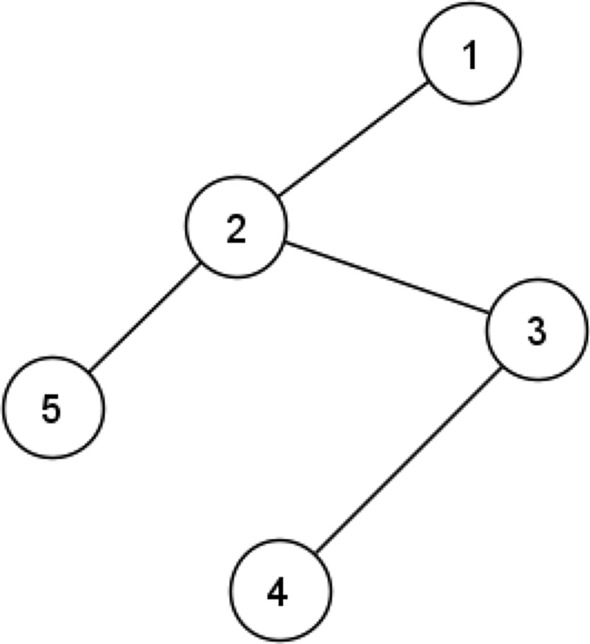
An example network (1).

A social network can be modelled as a graph *G*(*V*, *E*), where $V=\{v_1,v_2,v_3,\dots ,V_{\mid V\mid }\}$ denotes the set of nodes (users) and $\mid V\mid$ the number of nodes. The set $E \subset V \times V$ is a set of links indicating the relationships between nodes. If there is a link between two nodes $v_i$ and $v_j$, it is denoted by the edge $e_{ij}$, and the nodes are considered as neighbours or friends. Here, we use $\Gamma _i$ and $\Gamma _i^{(2)}$ to denote the set of first- and second-order neighbours of node $v_i$, i.e., $\Gamma _i=\{v_j \mid e_{ij} \in E \}$ and $\Gamma _i^{(2)}=\{v_k \mid e_{ij} \in E ,\, e_{jk} \in E ,\, e_{ik} \notin E\}$, respectively. The size of $\Gamma _i$ represents the degree of node $v_i$, i.e., $d_i=\mid \Gamma _i \mid$. Link prediction aims to estimate the probability of existence (or formation) of each of the non-existing links in the network in order to identify a set of missing or future links between the users. The set of non-existing links is denoted by $E^{N}=U-E$, where *U* is the universal set of the links in the network, i.e., $U=V \times V$. For example, consider the network shown in Fig. 1. In this network, $V=\{v_1,v_2,v_3,v_4,v_5\}$, $\mid V\mid =5$, $E=\{e_{12},e_{23},e_{25},e_{34}\}$. The set of non-existing edges is $E^{N}=\{e_{13},e_{14},e_{15},e_{24},e_{35},e_{45}\}$. The problem is to estimate the likelihood of formation for each of the links in $E^{N}$. In similarity-based methods, the likelihood of formation of a non-existing edge is estimated using a similarity score, which, for each pair of nodes, captures structural similarity of the nodes.

Different methods have been suggested to determine the similarity score, $S_{ij}$, between a pair of nodes $v_i$ and $v_j$. The number of common neighbours between two nodes is the best-known measure of similarity score. Based on this measure, the likelihood of formation of $e_{24}$ in Fig. 1 is higher than the likelihood of formation of $e_{45}$, because nodes 2 and 4 have one common neighbour whereas nodes 4 and 5 have no common neighbour, hence, $S_{24}=1>0=S_{45}$. Although computing the number of common neighbours is highly time-efficient, this measure cannot capture the similarity between two nodes accurately. Different measures^14–17^ have been proposed to improve the accuracy of this measure by combining the number of common neighbours with additional information. However, these measures also suffer from low accuracy. In fact, as will be demonstrated in the next section, relying on the number of common first-order neighbours between two nodes, similarity-based methods cannot capture well the topological similarity between a pair of nodes. Beyond direct relationships, latent relationships between two nodes, such as indirect connectivity, may be important in predicting future relationships. This observation motivates the work in this paper.

To build the argument of the paper, some real-world networks are first analysed to demonstrate the limitation of methods that rely on common first-order neighbours between the nodes as a similarity measure. To address this limitation, a measure is then proposed to take common second-order neighbours into account. Common second-order neighbours indicate a latent relationship between a pair of users. In this paper, we apply the Pearson correlation coefficient to capture the latent relationship between a pair of nodes. Based on the Pearson correlation coefficient, a new measure to estimate the similarity score for link prediction in social networks is proposed.

In the rest of the paper, the motivation for the proposed method is presented in the next section, followed by an overview of related work. Next, the proposed method is described in detail, followed by experimental evaluation. Finally, the paper is concluded with some suggestions for future work.

## Motivation

As suggested by Ke-ke et al.^18^, the number of common neighbours between a pair of nodes reveals structural similarity between the nodes and has a straight relationship with the link between the pair. However, as already mentioned, the number of common neighbours may be a simple and time-efficient method for link prediction, but it suffers from low accuracy and cannot provide comprehensive information to estimate the likelihood of link formation between the nodes. To demonstrate this, we examine nine different real-world networks including Zachary karate club (KRT)^19^, Hamsterster (HAM)^20^, Dolphins (DLN)^21^, US Airline (UAL)^22^, NetScience (NSC)^23^, Infectious (INF)^24^, Yeast (YST)^25^, email (EML)^26^ and KHN^27^ (detailed characteristics of these networks are summarized later in the paper, in Table 1). There are two key observations, which suggest that relying only on first-order neighbours is not an effective approach to estimate the likelihood of link formation.

**Figure 2:**
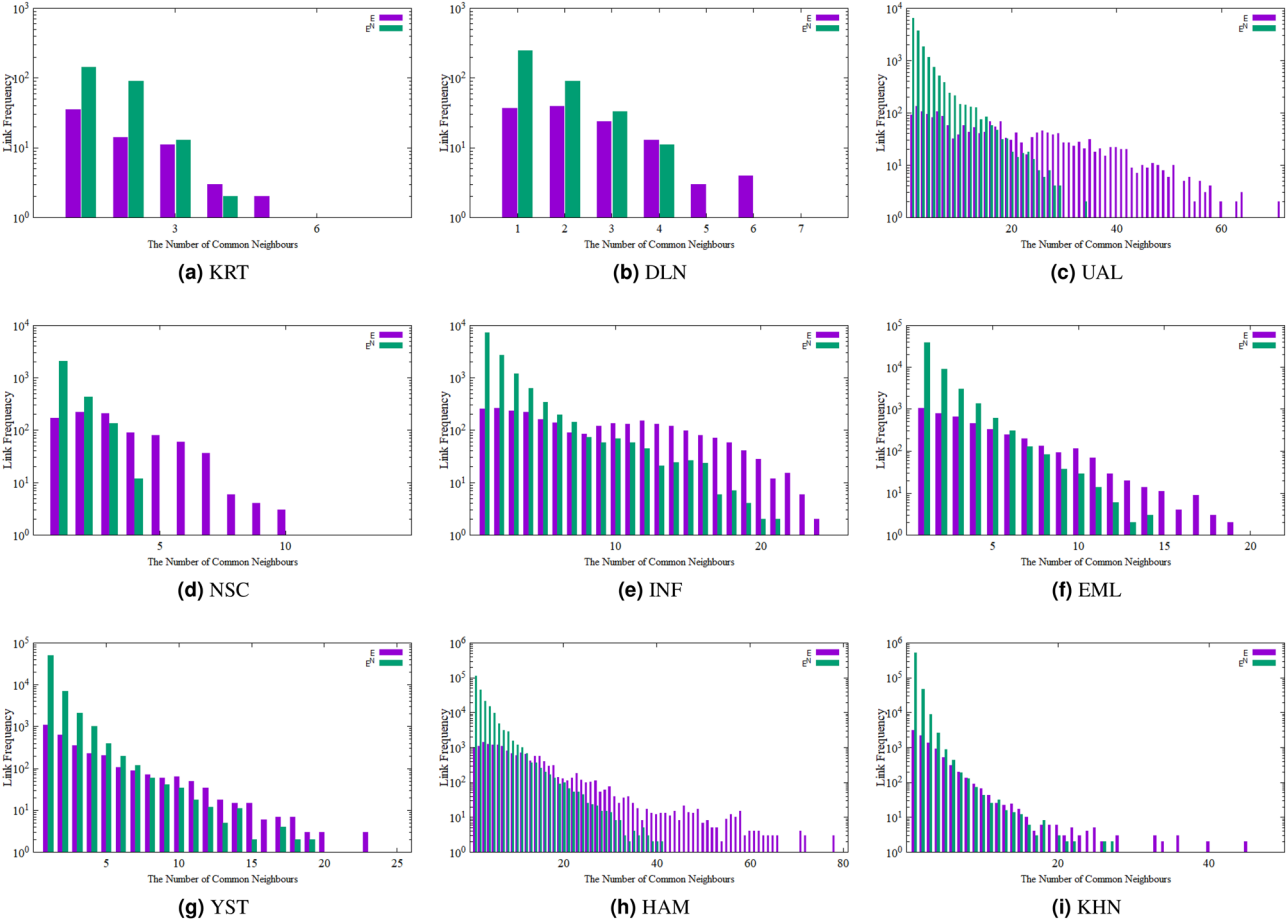
The frequency of links in *E* and $E^N$ with the same number of common neighbours.

- **Observation 1**: In real world-networks, a significant percentage of links may exist where the nodes connected by these links have no common neighbours. A quick check of the nine networks above reveals that this may indeed be a significant percentage. For example, 53.7% of the edges of the YST network have no common neighbour. In networks DLN, EML and KHN this value is 23.9%, 22.4% and 28.2% respectively. In the KRT network 14.1% of the links have no common neighbour. Finally, only in INF, NSC, UAL and HAM networks, this percentage is rather small: 4.9%, 4%, 3.1% and 3.8%, respectively. The suggestion is that considering common first-order neighbours may not always be a good predictor of future links. Depending on the network, methods whose prediction relies on common first-order neighbours alone may result in low accuracy.
- **Observation 2**: Sorting all existing links in a network (included in the set *E*), as well as all hypothetical links that may be formed between nodes without a link (defined as the set of non-existing links, $E^N$), by frequency for the same number of neighbours, we realize that there is a significant overlap. Consider, for example, Fig. 2. Although the set of (non-existing) links $E^N$ tends to have fewer common neighbours, on average, than the set *E*, there is a significant overlap between the two sets and, in some cases (say, around 8 common neighbours for the sets INF, EML, YST) the chance of an existing versus a non-existing link for that number of neighbours is essentially split in half. This is another suggestion that the number of common neighbours may not be a good indicator for link prediction.

In general, it appears that many links may exist between nodes that share no common neighbours at all, while, other nodes may share a large number of common neighbours without a direct link between them. Although it is true that various methods^14,16,17^ have been proposed to improve the accuracy of link prediction based on the number of common neighbours, the key limitation is that they still rely mostly on common first-order neighbours.

Based on the above, it seems there is scope to depart from common first-order neighbours. For example, two nodes may not have a common first-order neighbour, but they may still have many common second-order neighbours. That is to say, the number of common neighbours shows an explicit relationship between two nodes but there might be a relationship between two nodes which is not captured using common first-order neighbours. This kind of relationship is termed *latent relationship* in this paper. As suggested by observation 1 and 2, such latent relationship cannot be fully appreciated using simply common neighbours between the nodes. Considering the neighbourhood of two nodes may more accurately capture latent relationships between the nodes. For instance in the network shown in Fig. 1, nodes 4 and 5 have no common neighbours, but the correlation between their neighbours, i.e., nodes 2 and 3, may reveal a latent relationship between the two nodes, which correlates with the possibility of a future link between them. This kind of latent relationship should be considered for link prediction.

The above is what, essentially, motivates the research in this paper:

- **Hypothesis 1**: If there is no common neighbour between the nodes connected to a future link, but the nodes have a significant latent relationship, link formation can be predicted.
- **Hypothesis 2**: Considering latent relationships helps justify differences in existing and non-existing links between pairs of nodes that may still have the same number of common neighbours.

## Related work

There is a plethora of similarity-based methods for link prediction in the literature^4,7^. These methods essentially differ on what approach they use to estimate the similarity score between two nodes, which is then used to compute the likelihood of each non-existing link. Some methods estimate similarity based on neighbourhood, i.e., they are based on local structural information, while other methods may consider paths of different length between the nodes to take semi-local information into account or may first need to traverse the whole graph for global structural information and then estimate the likelihood of non-existing links based on this information.

Some of the most commonly used methods (which will also be used later for evaluation) are discussed below:

- Common Neighbours^8^: In this method, the number of common neighbours between each pair of nodes is considered as their similarity score. Thus, the common neighbour similarity score between the pair of nodes $v_i$ and $v_j$ is calculated according to Eq. (1).
  1 $$\begin{aligned} CN_{ij}=\mid \Gamma _i \cap \Gamma _j \mid \end{aligned}$$
- Preferential Attachment Index^10^: The degree of two nodes determines the likelihood of link formation. Thus, Eq. (2) is used to determine the similarity score between a pair of nodes $v_i$ and $v_j$.
  2 $$\begin{aligned} PA_{ij}=d_i \cdot d_j \end{aligned}$$
- Jaccard Index^11^: In this method, the similarity score between a pair of nodes $v_i$ and $v_j$ is calculated with the help of Eq. (3).
  3 $$\begin{aligned} JC_{ij}=\frac{\mid \Gamma _i \cap \Gamma _j \mid }{\mid \Gamma _i \cup \Gamma _j \mid } \end{aligned}$$
- Hub Promoted Index^28^: The ratio of the number of common neighbours to the minimum degree of nodes $v_i$ and $v_j$ is defined as the similarity measure. The similarity score of these nodes is calculated with the help of Eq. (4).
  4 $$\begin{aligned} HPI_{ij}=\frac{\mid \Gamma _i \cap \Gamma _j \mid }{min \{d_i,d_j\}} \end{aligned}$$
- Common Neighbours Degree Penalization^15^: Penalization of common neighbours is considered in this method. The number of common neighbours for each pair of common neighbours of the two nodes is taken into account for this purpose. Then, the similarity score of nodes $v_i$ and $v_j$ is calculated using Eq. (5), where $CN_z^{(2)}=\{\Gamma _z \cap \Gamma _i \cap \Gamma _j\} \cup \{v_i, v_j\}$.
  5 $$\begin{aligned} CNDP_{ij}=\sum _{v_z \in \Gamma _i \cap \Gamma _j} \mid CN_z^{(2)}\mid (d_z^{\, -\beta C}) \end{aligned}$$
- Node-Coupling Clustering^17^: In this method, the clustering coefficient is used to determine the contribution of each common neighbour and the similarity between each pair of nodes. The similarity score between $v_i$ and $v_j$ is calculated using Eq. (6), where $C_z$ is the clustering coefficient of node $v_z$.
  6 $$\begin{aligned} NCC_{ij}=\sum _{v_n \in \Gamma _i \cap \Gamma _j} \frac{\sum _{v_z \in CN_n^{(2)}}(\frac{1}{d_z}+C_z)}{\sum _{v_w \in \Gamma _n}(\frac{1}{d_w}+C_w)} \end{aligned}$$
- Parameterized Algorithm^16^: In this method, the number of common neighbours and the closeness of two nodes are both taken into account to estimate the similarity between a pair of nodes. The parameterized similarity score between $v_i$ and $v_j$ is calculated by Eq. (7), where $\alpha$ is a tunable parameter and $d_{ij}$ is the shortest distance between nodes $v_i$ and $v_j$.
  7 $$\begin{aligned} CCPA_{ij}=\alpha (\mid \Gamma _i \cap \Gamma _j \mid )+ (1-\alpha ) \frac{\mid V \mid }{d_{ij}} \end{aligned}$$
- Higher-Order Path Index^29^: Based on the common neighbours, the significance of paths between two nodes is taken into account to propose an iterative method. Summing up the significance of the paths between two nodes determines the likelihood of link formation between them. For this purpose, the significance of a path of length 2 between nodes $v_i$ and $v_j$ is calculated using Eq. (8).
  8 $$\begin{aligned} S_{ij}=\sum _{v_n \in \Gamma _i \cap \Gamma _j}\frac{1}{d_z} \end{aligned}$$
  The significance of paths of length $l>2$ between nodes $v_i$ and $v_j$ is calculated based on the significance of its constituent edges using Eq. (9).
  9 $$\begin{aligned} S_{ij}=\sum _{k=3}^{l-2} f_1 \cdot f_2 \cdot \alpha ^{l-2}, \end{aligned}$$
  where $f_1$ and $f_2$ denote the significance of the constituent edge and the significance of the path of previous iteration, and $\alpha$ is a tunable parameter.

Apart from these methods, various other local and semi-local methods have been used to estimate similarity between a pair of nodes. Local methods include: Adamic Adar index^30^, Sorensen index^10^, resource allocation index^31^, node clustering coefficient^32^, node and link clustering coefficient^33^, heterogeneity index^34^ and tie connection strength index^35^. Semi-local methods, which estimate the likelihood of link formation between a pair of nodes on the basis of the paths between them, include: effective paths index^36^, significant paths index^37^, penalizing non-contribution links index^38^, local paths^39^ and friend link^40^.

In this paper, a novel method is proposed, which goes beyond the number of common neighbours by taking into account local information from both first- and second-order neighbourhood of the nodes.

## A novel method for link prediction based on latent relationships

In this section, we propose a novel method for similarity-based link prediction, which we call Direct-Indirect Common Neighbours (DICN). This method takes into account latent relationships between nodes as will be described next. The idea is first to estimate the impact of common second-order neighbours between each pair of nodes. Then, this is combined with the impact of common first-order neighbours to estimate the similarity between the pair.

In order to determine the impact of common second-order neighbours, a neighbourhood vector $N_i$ is first defined for each node *i* with $\mid V\mid$ entries as in Eq. (10). The *z*th entry of this vector corresponds to node *z*. When $z=i$, we set $N_i[i]=d_i$, that is, the degree of node *i*. If node *z* is a second-order neighbour of node *i* (in this case, by definition, node *z* is not a first-order neighbour of node *i*), we set the corresponding vector entry, $N_i[z]$, to $CN_{iz}$ (see Eq. (1)), whereas, if node *z* is a first-order neighbour of node *i*, we add 1 to this quantity. Finally, if node *z* is not a first- or second-order neighbour of node *i*, they do not have any common neighbour, so $N_i[z]=0$.

10 $$\begin{aligned} N_i[z]_{\, z=1,2, \dots ,\mid V\mid }\quad = {\left\{ \begin{array}{ll} d_i &{} if\,z=i \\ CN_{iz} &{} if\, v_z \in \Gamma _i^{(2)} \\ CN_{iz}+1 &{} if v_z \in \Gamma _i \\ 0 &{} \text {otherwise} \end{array}\right. } \end{aligned}$$

In order to estimate the likelihood of link formation between nodes $v_i$ and $v_j$, the union neighbourhood set, $UN_{ij}$, for these nodes is calculated using Eq. (11).

11 $$\begin{aligned} UN_{ij}=\{z \mid (N_i[z]>0) \;Or \; (N_j[z]>0)\} \end{aligned}$$

Greater correlation between the union neighbourhood set, $UN_{ij}$, of the vectors $N_i$ and $N_j$ indicates higher structural similarity between nodes *i* and *j*. Thus, the correlation coefficient between the union neighbourhood set of the vectors is then calculated to determine the correlation between two nodes. We use Pearson correlation coefficient for this purpose, thus, the correlation between the union neighbourhood set of the vectors $N_i$ and $N_j$ is calculated using Eq. (12).

12 $$\begin{aligned} Corr_{ij}=\frac{\sum _{z\in UN_{ij}} (N_i[z]-\overline{N_i})\, (N_j[z]-\overline{N_j})}{\sqrt{\sum _{z\in UN_{ij}}(N_i[z]-\overline{N_i})^2} \, \sqrt{\sum _{z\in UN_{ij}}(N_j[z]-\overline{N_j})^2}} \end{aligned}$$

In Eq. (12), $\overline{N_i}$ is the mean of the values in the union neighbourhood set of vector $N_i$; it is calculated using Eq. (13).

13 $$\begin{aligned} \overline{N_i}=\frac{\sum _{z\in UN_{ij}}N_i[z]}{\mid UN_{ij}\mid } \end{aligned}$$

**Figure 3:**
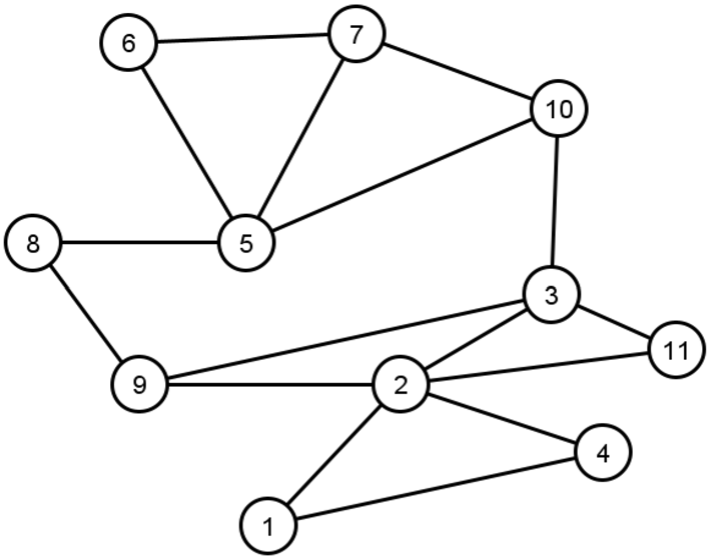
An example network (2).

In our method, two nodes that do not have common neighbours may still have significant structural similarity. Thus, a relationship may be detected through correlation between their neighbours. Take, for example, the links $e_{31}$ and $e_{38}$ in the network shown in Fig. 3. Based on Eq. (12), nodes 3 and 1 have higher structural similarity, because $Corr_{38}\cong 0.32$ and $Corr_{31}\cong 0.01$. When the neighbours of two nodes are highly correlated a latent relationship between the nodes is implied. Thus, in Eq. (12), greater correlation between two nodes shows higher indirect similarity between the nodes and formation of a link between them can be regarded as likely. Direct similarity between two nodes is calculated based on the number of common first-order neighbours. We combine indirect and direct similarity in Eq. (14) to calculate the Direct-Indirect Common Neighbours (DICN) similarity score of nodes *i* and *j*.

14 $$\begin{aligned} DICN_{ij}= (1+CN_{ij}) (1+Corr_{ij}) \end{aligned}$$

Pseudo-code to implement the proposed method is shown in Algorithm 1. In lines 1–5 of the algorithm, the neighbourhood vector, $N_i$, for each node $v_i$ is calculated. The likelihood of formation of each non-existing link between nodes $v_i$ and $v_j$ is calculated in lines 6–10, whereas the union neighbourhood set and the indirect similarity between the nodes are calculated in lines 7 and 8, respectively. The link formation likelihood is computed in line 9 resulting in the *DICN* similarity score. 
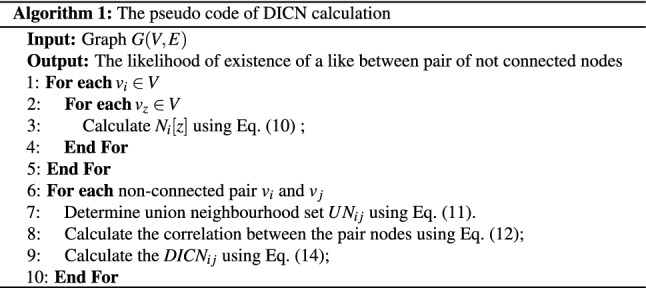


**Example:** Take the network in Fig. 3, as an example. In this network $\mid V\mid =11$. Vectors $N_2$ and $N_5$ are calculated as follows:

$$\begin{aligned} N_2= & {} \{2,5,3,2,0,0,0,1,2,1,2\} \\ N_5= & {} \{0,0,1,0,4,2,3,1,1,2,0\} \end{aligned}$$

Furthermore, $UN_{25}=\{1,2,3,4,5,6,7,8,9,10,11\}$. The indirect similarity between $v_2$ and $v_5$ is calculated below:

$$\begin{aligned} Corr_{25}=\frac{\sum _{z\in UN{25}} (N_2[z]-\overline{N_2})\, (N_5[z]-\overline{N_5})}{\sqrt{\sum _{z\in UN{25}}(N_2[z]-\overline{N_2})^2} \, \sqrt{\sum _{zz\in UN{25}}(N_5[z]-\overline{N_5})^2}}\cong -0.74 \end{aligned}$$

Finally, the DICN similarity score between the nodes is given by:

$$\begin{aligned} DICN_{25}=(1+0)(1+(-0.74))=0.26 \end{aligned}$$

## Experimental results

### Setting

In order to evaluate the performance of the proposed DICN method, this method and another 8 representative methods from the literature were implemented in Java and executed on a PC with an i5 2.3 GHz processor and 8 MB memory. The eight methods used for comparison are: Common Neighbours (CN)^8^, Preferential Attachment Index (PA)^10^, Jaccard Index (JC)^11^, Hub Promoted Index (HPI)^28^, Common Neighbours Degree Penalization (CNDP)^15^, Node-coupling Clustering (NCC)^17^, Parameterized Algorithm (CCPA)^16^ and Significance of Higher-Order Path Index (SHOPI)^29^.

Nine different real-world networks with a variety of features were used in the experiments. Zachary karate club (KRT)^19^ and Hamsterster (HAM)^20^ are social networks. Dolphins (DLN)^21^ is an animal network. US Airline (UAL)^22^ is an airport traffic network. NetScience (NSC)^23^ and KHN^27^ are co-authorship networks. Infectious (INF)^24^ is a network of face-to-face contacts in an exhibition. Yeast (YST)^25^ is a biological network. U. Rovira i Virgili email (EML)^26^ is an email communication network. Specific characteristics for each of the networks are shown in Table 1.

**Table 1:** Characteristics of the nine networks used in the experiments showing the number of nodes ($\mid V\mid$), the number of edges ($\mid E \mid$), average clustering coefficient ($\langle C\rangle$), average degree ($\langle d\rangle$) and degree assortativity (*r*).

Network	$\mid V\mid$	$\mid E\mid$	$\langle C\rangle$	$\langle d\rangle$	*r*
KRT	34	78	0.26	4.59	−0.4756
DLN	62	159	0.31	5.13	−0.0436
UAL	332	2126	0.63	12.81	−0.2079
NSC	379	914	0.74	4.82	−0.0817
INF	410	2765	0.46	13.49	0.2258
EML	1133	5451	0.22	9.62	0.0782
YST	2,284	6646	0.13	5.82	−0.0991
HAM	2,426	16,630	0.54	13.71	0.0474
KHN	3,772	12,718	0.25	6.74	−0.1205

We follow an evaluation strategy, which is in line with the evaluation strategies used in other related work^16,17^. For each network, the set of existing edges, *E*, is randomly divided into two sets: the set of training edges $E^T$ and the set of test edges $E^P$, where $E^T \cap E^P=$ and $E^T \cup E^P=E$. We randomly select $\beta$ percent of edges as $E^T$ and the remaining, $1-\beta$ percent of edges, as $E^P$. To increase the confidence of the obtained results, the process is repeated 15 times and the average of the obtained results is reported in each experiment. The metric *Area Under the receiver operating characteristic Curve* (*AUC*), widely applied in the relevant literature^1^, is used to assess the accuracy of methods. The *AUC* is computed by picking an edge from $E^P$ and an edge from the set of non-existing edges, $E^N$, and calculating the similarity score between the pair of nodes connected to each of the edges. This process is repeated *n* times and *AUC* is calculated using Eq. (15).

15 $$\begin{aligned} AUC=\frac{n_1+\frac{1}{2}n_2}{n} \end{aligned}$$

In Eq. (15), $n_1$ is the number of times when the similarity score of the nodes connected by the edge picked from the set $E^P$ is higher than the similarity score of the nodes connected by the edge picked from the set $E^N$, and $n_2$ is the number of times when the two similarity scores are equal. With respect to the value of *n*, in our experiments we always compare every pair of links in $E^P$ and $E^N$. This means that $n=|E^P| \cdot |E^N| = (1 - \beta /100) \cdot |E| \cdot (\frac{|V| \cdot (|V| -1)}{2}-|E|)$, where $\beta$ is the percentage of edges in the training set, $E^T$. The value of *AUC* is between [0, 1], where a higher value shows higher accuracy.

**Figure 4:**
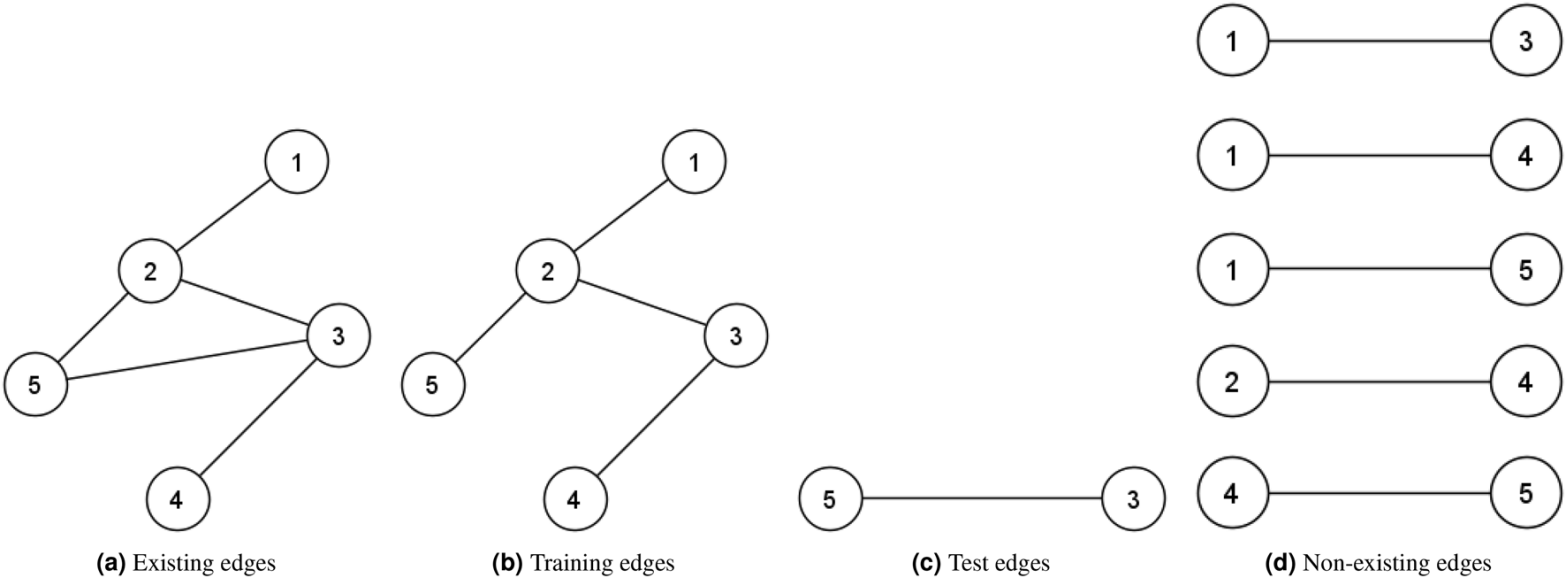
A simple example of the different sets or AUC calculation.

We highlight the process of calculating AUC using an example. Consider the network shown in Fig. 4a and assume $\beta =80\%$. This network has 5 edges which, as shown in Fig. 4b,c, are randomly divided into a training edges set and a test edges set with 4 edges and 1 edge, respectively. The non-existing edges set for this network is shown in Fig. 4d. In order to calculate AUC in this example the likelihood of formation for the test edge $e_{35}$ must be compared to non-existing edges $e_{13}$, $e_{14}$, $e_{15}$, $e_{24}$ and $e_{45}$. Applying Eq. (14), $DICN_{35}=2.5$, $DICN_{13}=2.5$, $DICN_{14}=0.59$, $DICN_{15}=2.0$, $DICN_{24}=2.82$ and $DICN_{45}=0.59$. Thus, $n_1=3$ and $n_2=1$ and $AUC=\frac{3+\frac{1}{2}\times 1}{5}=0.7$.

### Results

Four different experiments are performed. Their objective is, respectively, to: (1) assess the accuracy of DICN when compared to other methods; (2) assess the robustness of DICN, with different sizes of training data; (3) and (4) validate Hypothesis 1 and 2 described earlier in the motivating section.

#### Experiment 1

In the first experiment, we consider a value of $\beta$ equal to 80, as this is a value commonly used in other related experiments^9,16^. Then, for each of the nine methods and each of the nine networks, we calculate the value of *AUC*. The results are shown in Table 2. It can be seen that in eight of the nine networks, DICN outperforms all other methods. Even for the UAL network, DICN’s accuracy is very close to the best accuracy. As it relies on both the number of common neighbours and the correlation between the neighbours, DICN takes into account both direct and indirect similarity between the nodes which leads to better accuracy in distinguishing the links in $E^P$ and $E^N$ than other methods.

**Table 2:** *AUC* of different methods in different networks. The best result in each network is shown with bold face.

Network	CN	PA	JC	HPI	NCC	CNDP	CCPA	SHOPI	DICN
KRT	0.6884	0.6976	0.6884	0.6405	0.7044	0.7336	0.6995	0.7328	**0**.**7654**
DLN	0.7298	0.6072	0.7298	0.6303	0.7300	0.7276	0.7570	0.7285	**0**.**7943**
UAL	0.9289	0.8852	0.8913	0.7802	**0**.**9416**	0.9410	0.9195	0.9293	0.9367
NSC	0.9166	0.6171	0.9134	0.8302	0.9194	0.9195	0.8922	0.9050	**0**.**9747**
INF	0.9279	0.7009	0.9297	0.9094	0.9309	0.9278	0.9483	0.9292	**0**.**9553**
EML	0.8186	0.7767	0.8158	0.7465	0.8187	0.8180	0.8701	0.8729	**0**.**8932**
YST	0.6866	0.7737	0.6798	0.5319	0.6866	0.6869	0.7892	0.7547	**0**.**8278**
HAM	0.9520	0.8467	0.9450	0.8805	0.9553	0.9534	0.9617	0.9531	**0**.**9725**
KHN	0.7786	0.8090	0.7592	0.6651	0.7833	0.7840	0.8253	0.8509	**0**.**8839**

**Figure 5:**
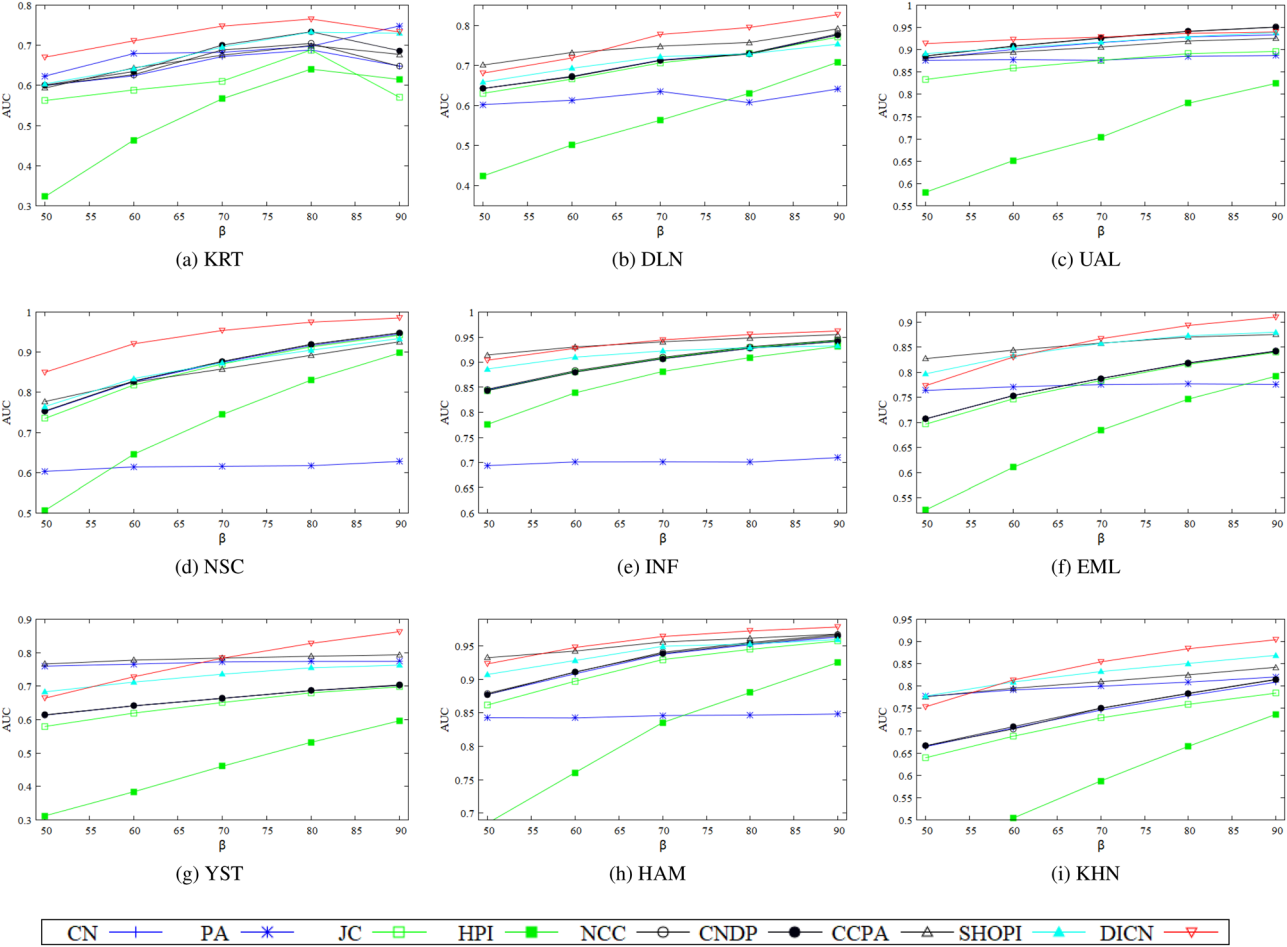
The impact of varying the training set ratio on *AUC* for different methods.

#### Experiment 2

In the next experiment, the robustness of the different methods with respect to the size (that is, the value of $\beta$) of the training set $E^T$, is evaluated. For this purpose, the value of $\beta$ is varied from 50 to 90 in steps of 10, a range where some reasonably good accuracy is expected and is in line with other studies^9^. The accuracy of different methods for each value of $\beta$ is calculated by *AUC*. As all networks tend to follow a similar trend where higher values of $\beta$ tend to increase accuracy, we show results in Fig. 5. Although, for small values of $\beta$, DICN does not have the best accuracy for some networks, this method is consistently best when the value of $\beta$ is 70 or higher in seven of the nine networks. This is because, when the training set is smaller it is harder to detect the latent relationship between the nodes due to the lower correlation between them. So DICN may not be so accurate in networks with a relatively small training set. However, in the presence of a large training set the correlation between the nodes is detected more accurately and the latent relationship is estimated by DICN more accurately. It is also interesting to observe that in some networks DICN outperforms all other methods significantly, something that could be investigated further to document the advantages of DICN.

**Table 3:** Ability of methods to distinguish links between nodes with no common neighbours. The best result in each network is shown with bold face.

Network	PA	CCPA	SHOPI	DICN
KRT	0.7519	0.7211	0.6487	**0**.**8319**
DLN	0.4954	**0**.**7211**	0.6866	0.7028
UAL	0.6833	0.5900	**0**.**8091**	0.7979
NSC	0.6766	0.6750	0.6035	**0**.**8471**
INF	0.4506	0.8072	**0**.**8404**	0.7979
EML	0.6637	0.7132	0.7407	**0**.**7595**
YST	**0**.**7755**	0.7653	0.6276	0.7609
HAM	0.7296	0.7803	0.7326	**0**.**7831**
KHN	0.7798	0.7278	0.7326	**0**.**8001**

#### Experiment 3

This experiment is dedicated to the validation of Hypothesis 1, which relates to the ability of the methods to distinguish links between nodes with no common neighbours. To do so, for each of the nine networks we take the set of test edges, $E^P$ and the set of non-existing edges, $E^N$. From these two sets, we select those edges that connect nodes that have no common neighbours and the degree of these nodes is greater than 1. Then we calculate the similarity score for each of these edges for our proposed method DICN and all other methods. We note that, with the exception of PA, CCPA and SHOPI, all other methods will result in a similarity score of zero, as the edges we selected are between nodes that have no common neighbour; hence, these methods are omitted for further analysis. The *AUC* of PA, CCPA, SHOPI and DICN methods is shown in Table 3. It can be seen that DICN is more accurate than other methods when distinguishing links between nodes with no common neighbours for five of the nine networks, while it has an accuracy very close to the best for the remaining four networks. In this experiment, by default the value of direct similarity in Eq. (14) is zero for all compared edges. Still, DICN can accurately distinguish the test and non-existing edges. Once again, this experiment suggests that calculating the correlation between neighbourhood vectors provides a good accuracy to detect indirect similarity between nodes when there are no common neighbours between them.

**Table 4:** Ability of methods to distinguish links between nodes with the same number of common neighbours. The best result in each network is shown with bold face.

Network	NCC	CNDP	CCPA	SHOPI	DICN
KRT	0.5637	0.6292	0.5594	**0**.**6919**	0.6728
DLN	0.4978	0.4953	**0**.**6510**	0.6157	0.6508
UAL	0.7161	0.7192	0.5153	**0**.**7921**	0.5684
NSC	0.5616	0.5689	0.6308	0.6432	**0**.**7815**
INF	0.5275	0.5335	0.7490	0.7598	**0**.**7712**
EML	0.5055	0.5089	0.6975	0.7331	**0**.**7495**
YST	0.5009	0.5019	**0**.**7613**	0.6278	0.7595
HAM	0.5381	0.5425	0.7352	0.7373	**0**.**7483**
KHN	0.5251	0.5284	0.7066	0.7682	**0**.**7829**

#### Experiment 4

This experiment is dedicated to validation of Hypothesis 2, which relates to assessing the ability of the methods to distinguish links between nodes with the same number of common neighbours. To do so, for each of the nine networks we take again the set of test edges, $E^P$ and the set of non-existing edges, $E^N$. From these two sets, we select the edges that connect nodes with the same number of common neighbours. Then we calculate the similarity score for each of these edges using our proposed method DICN, and the best performing methods from Experiment 2: NCC, CNDP, CCPA and SHOPI. The *AUC* of each method is shown in Table 4. Once again, the ability of DICN to consider latent relationships leads to higher accuracy in five of the nine networks. In the KRT, DLN and YST networks, DICN has results that are close to the best method. Only in the UAL network the NCC, CNDP and SHOPI methods significantly outperform DICN. Overall, the results obtained in this experiment confirm that assessing correlation using a neighbourhood vector for nodes is an accurate way to distinguish the test and non-existing edges of nodes with an equal number of common neighbours.

## Conclusion

The prediction of future links and the identification of missing links have attracted significant research in social networks analysis. Different methods have been proposed for it, many of which are based on the number of common neighbours. The idea behind this paper has been that latent relationships between the nodes are not captured by the number of common neighbours. Thus, to take into account such latent relationships, a correlation-based measure was proposed and its accuracy was compared to other related methods, giving superior accuracy results. Further work can look into more elaborate experimentation and networks with varying characteristics, including directed and weighted networks. In addition, the definition of latent relationship can be expanded beyond second-order relationships, for example including correlation with the number of paths between the nodes or global properties, such as centrality of the nodes, and so on.
